# Adverse Reaction to COVID-19 mRNA Vaccination in a Patient With VEXAS Syndrome

**DOI:** 10.7759/cureus.23456

**Published:** 2022-03-24

**Authors:** Giulio Ciprian

**Affiliations:** 1 Internal Medicine, Roger Williams Medical Center, Providence, USA

**Keywords:** uba1, acute rheumatology, covid-19, multiple autoimmune syndrome, vexas

## Abstract

VEXAS (vacuoles, E1 enzyme, X-linked, autoinflammatory, somatic) syndrome is a rare genetic disorder originating from a somatic mutation in the hematopoietic stem cells. This syndrome was first described in 2020 and carries many clinical features that other conditions cannot explain. Widespread autoinflammation is the primary process the disease presents, with high morbidity and mortality in those who show signs of bone marrow failure. Treatment is complex, and response to current therapies is poor. Long-term prognosis carries a mortality of 50%. In addition, the advancement of new-generation messenger ribonucleic acid (mRNA) vaccines raises concerns about their safety in this population since it could trigger a vaccine-related autoimmune response. This case describes the hospital course of a male in his 50s exhibiting an unexplained cutaneous reaction to an mRNA COVID-19 vaccine. He was later diagnosed with VEXAS syndrome based on symptoms presentation and diagnostic workup.

## Introduction

In recent years, a new autoimmune disorder was discovered. The syndrome is called VEXAS (vacuoles, E1 enzyme, X-linked, autoinflammatory, somatic). Its name outlines the different features portrayed by this condition. While the relatively new nature of the disease, the literature has described the various manifestations, including cutaneous and hematologic involvement. To begin with, VEXAS is caused by a somatic mutation in the *UBA1* (ubiquitin-like modifier activating enzyme 1) gene located on the p arm of the X chromosome [1-2]. The function of the enzyme E1 coded by this gene is to catalyze the first step in ubiquitylation that serves as a marker for cellular protein degradation. Moreover, this gene plays a central role in regulating the human cell since it is one of the only two enzymes involved in this process, the second one being the *UBA6* [3]. The ubiquitylation process has a significant role in the cell cycle, such as regulating endocytosis, signal transduction, apoptosis, and protein folding through the NEDD8 pathway [4]. This gene has been previously associated with spinal muscular diseases, including *AMCX1* (arthrogryposis multiplex congenital X-linked type 1) and spinal muscular atrophy [5]. More and more patients are diagnosed retrospectively with VEXAS syndrome, with an age distribution between 40 and 80 years and a median onset of symptoms in the late 50s to mid-60s [6-7]. Hematopoietic stem cells are the main target of this mutation, leading to an activation of the innate immune system that emerges clinically in many forms. Cutaneous presentations include polychondritis, vasculitis, and neutrophilic dermatoses [7]. Hematological features are consistent with macrocytic anemia, thrombocytopenia, myelodysplastic syndrome (MDS), multiple myeloma, and monoclonal gammopathy of undetermined significance (MGUS). The overall systemic inflammation caused by this autoimmune condition can lead to multi-organ failure and thrombotic events, including venous and arterial thromboembolism. Diagnostic workup includes bone marrow biopsy (hypercellular with vacuoles within erythroid and myeloid precursors), skin biopsy with cluster of differentiation (CD) 163+ myeloid cells as well as perivascular neutrophils with leukocytoclasis. However, deoxyribonucleic acid (DNA) sequencing is the only test able to confirm a definitive diagnosis by identifying the *UBA1 *gene mutation. This report describes the case of a patient who presented to the intensive care unit (ICU) with distributive shock and a cutaneous reaction after receiving a new mRNA vaccine against the SARS-CoV-2 virus. Initially, after undergoing all three doses, including the booster, the patient developed a tense bulla with erythema and extreme tenderness at the injection site on the right arm. After extensive workup, the patient was diagnosed with VEXAS syndrome through DNA testing. This report will illustrate this patient's clinical history, including how our team managed the hospital course and finally diagnosed him with this sporadic genetic disease.

## Case presentation

**Figure 1:**
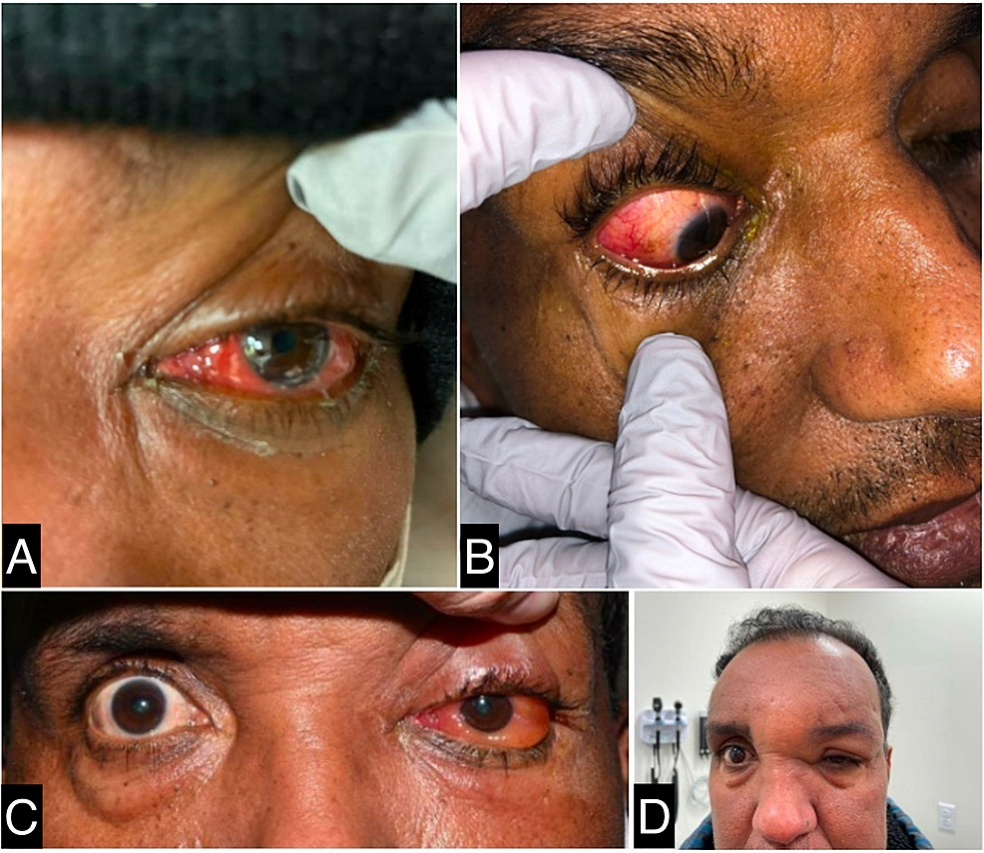
Images A, B, and C show ocular inflammation in both eyes at different times. Image D: shows scleritis with associated facial swelling.

**Figure 2:**
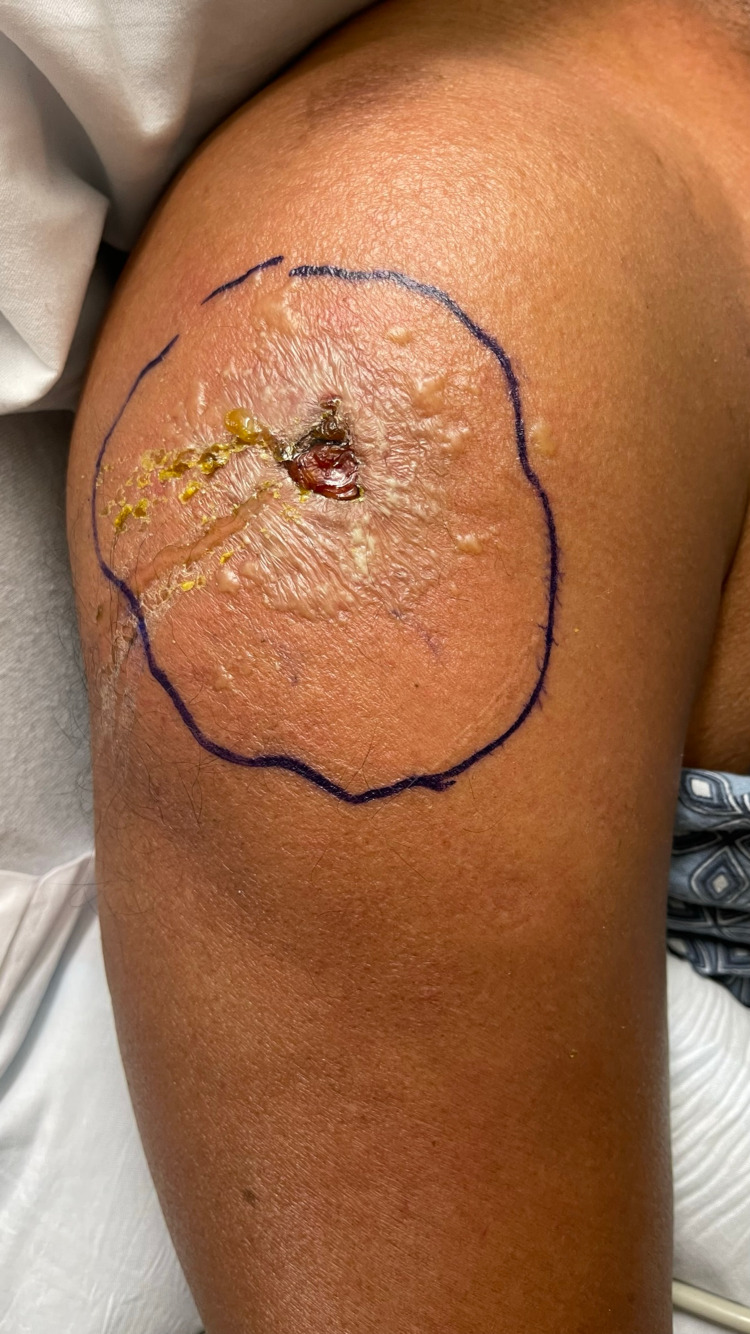
Ruptured bulla on the right arm at the injection site.

The patient is a 56-year-old Spanish-speaking male with a medical history significant for arthritis, polychondritis, pancytopenia (persisting transfusion-dependency), splenomegaly, splenic infarctions on Eliquis®, orbital inflammatory syndrome of unclear etiology (Figure 1), and nonischemic heart failure with reduced ejection fraction (HFrEF) (ejection fraction of 35%). He presented to the emergency department (ED) for fever, nausea, vomiting, and diarrhea of one-day duration. He received the Moderna COVID-19 booster, after which he developed significant tenderness along with swelling at the injection site (Figure 2). Shoulder pain was 10/10 in intensity, with limited range of motion at the joint. He denied any hematochezia or hematemesis. Moreover, he stated that he had similar symptoms after his second COVID-19 vaccination six months prior to presentation. He denied any use of alcohol or drugs, or smoking of cigarettes. He, however, recently traveled to the Caribbean, where he was hospitalized and received multiple blood transfusions; sick contacts were unknown at that time. He denied any family or personal history of sickle cell disorder.

**Figure 3:**
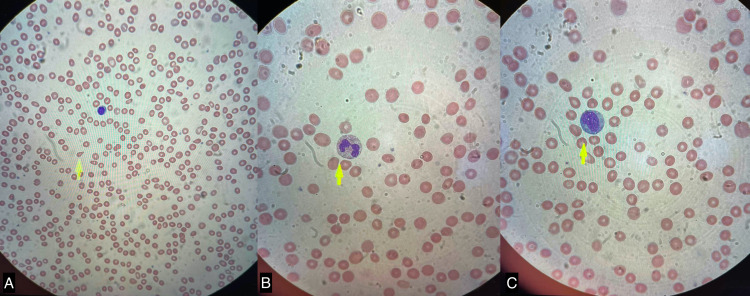
Image A shows teardrop RBC on peripheral smear. Images B and C show vacuoles within the nucleated cells. RBC, red blood cells

Hematological workup performed before admission for pancytopenia was consistent for a blood smear with normocytic normochromic red blood cells (RBCs), teardrop cells, and rare schistocytes (Figure 3). Platelets had normal morphology, and white blood cell (WBC) analysis did not show any evidence of premature forms. He underwent a bone marrow biopsy, which showed a hypercellular bone marrow with relative myeloid hyperplasia and megakaryocyte dysplasia (relative expansion of the gamma delta T-cell subpopulation associated with clonally expanded rearrangement of the T-cell receptor beta [TRB] locus) with vacuoles in myeloid and erythroid cells. Furthermore, the patient was evaluated by the pulmonology service because of mediastinal lymphadenopathy noticed on a computed tomography (CT) scan. Initially, endobronchial ultrasound (EBUS) guided biopsy was considered; however, it was later deferred due to scanty lymph nodes and the high risk of general anesthesia given his advanced heart failure. Outpatient cardiac magnetic resonance imaging (MRI) showed no evidence of gadolinium enhancement within the myocardium to suggest infiltrative processes such as sarcoidosis. Prior MRI of the orbits was consistent with an intraconal/retro-orbital fat tracking proximally along the optic nerve sheath to the orbital apex and right scleritis with right superior rectus muscle involvement. Six months prior, the patient underwent a trial of methotrexate for indeterminate connective tissue disease without improvement of his symptoms. He also received multiple courses of prednisone for his relapsing and remitting pattern of scleritis and polychondritis with partial and temporary response to increasing doses of steroids.

**Figure 4:**
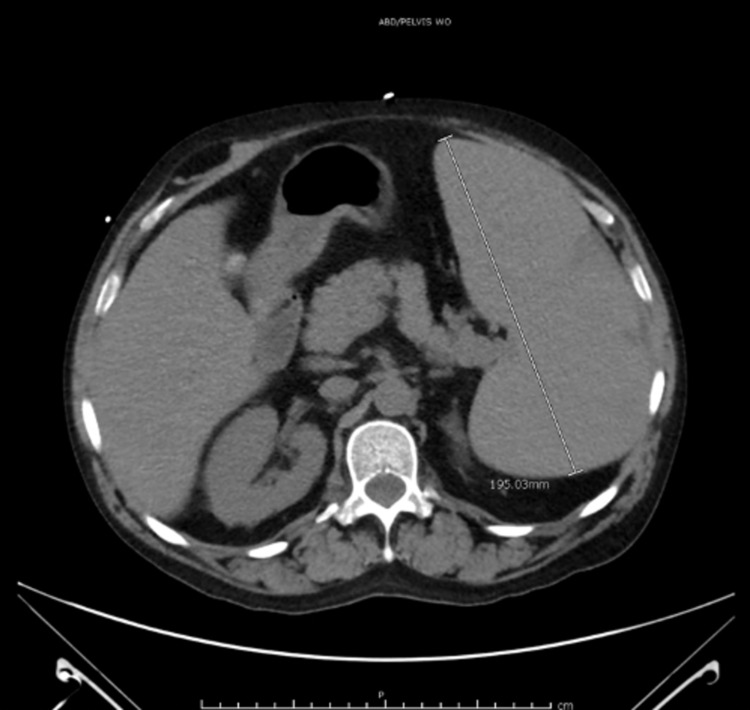
CT of the abdomen showing splenomegaly up to 19.5 cm with hypodensities at the periphery consistent with splenic infarcts.

Initial labs on admission were consistent with leukocytosis, thrombocytopenia, and anemia with macrocytosis (Table 1). The hemolytic panel, including direct and indirect antiglobulin testing, haptoglobin, and lactate dehydrogenase (LDH), were unremarkable and not suggestive of hemolytic anemia. Inflammatory markers, including C-reactive protein (CRP) and erythrocyte sedimentation rate (ESR), were elevated (Table 1). CT of his abdomen showed worsening splenomegaly with splenic infarcts (Figure 4). An ultrasound of his extremities ruled out any deep venous thrombosis. The patient was constantly hypotensive, unresponsive to intravenous fluids resuscitation, and required vasopressor. Broad-spectrum antibiotics were initiated for suspected septic shock; however, pan-cultures did not yield any pathogen despite an elevated procalcitonin (Table 1), making distributive-anaphylactic shock the most likely cause of his hypotension. He was also started on stress dose steroids for his low blood pressure with hydrocortisone, which was later tapered to prednisone. He also received one unit of packed RBCs and two units of platelets during this admission.

**Table 1:** Initial labs on CBC CBC, complete blood count; WBC, white blood cells; Hgb, hemoglobin; MCV, mean corpuscular volume; CRP, C-reactive protein; ESR, erythrocyte sedimentation rate

Lab	Results	Reference Values	Units
WBC	11.3	4-11	× 10^3^/µL
Hgb	11.8	14.0-18.0	g/dL
Platelets	47	150-450	× 10^3^/µL
MCV	112.9	75-100	fL
procalcitonin	155.87	0.05-0.5	ng/mL
CRP	309.80	0.00-7.30	mg/L
ESR	41	0-15	mm/h

Autoimmune workup revealed elevated levels of B2GP1 (beta-2 glycoprotein 1) immunoglobulin (Ig) M antibody, which raised the concern for APLS (antiphospholipid syndrome) as the cause of the unprovoked splenic infracts. Previous records also indicated consistent workup for single-positive APLS and splenic thrombosis. Lupus anticoagulant and anticardiolipin antibodies were negative (Table 2). While in the hospital, however, Eliquis was discontinued due to thrombocytopenia and concerns for bleeding. Additional labs were non-suggestive of any other underlying autoimmune process (Table 2). Antibiotics were gradually de-escalated, and his need for pressors decreased.

**Table 2:** Labs presenting different types of immunologic and infectious workup performed on the patient B2GP1 IgM Ab, beta-2 glycoprotein 1 immunoglobulin M antibody; ANA, anti-nuclear antibodies; RF, rheumatoid factor; anti-CCP IgG Ab, anti-citrullinated protein immunoglobulin G antibodies; ANCA, anti-neutrophil cytoplasmic antibodies; HLA, human leukocyte antigen; C1Q, complement component 1Q binding; ACE, angiotensin-converting enzyme

Lab	Results	Reference values	Units
B2GP1 IgM Ab	76.8	<20	U/mL
Lupus anticoagulant	Negative	Negative	
Anticardiolipin IgM Ab	19	<20	GPL-U/mL
ANA	Non-reactive	Non-reactive	
RF	22	0-30	IU/mL
anti-CCP IgG Ab	22	0-30	U
ANCA	Negative	<1:20	
HLA-B27	Negative	Negative	
Treponema pallidum IgG and IgM	0.2	0.0-0.8	AI
Lyme Ab	0.82	0.00-0.091	I
QuantiFERON gold	Negative	Negative	
C1Q binding assay	<1.2	<25.2	mcg Eq/mL
C1 esterase inhibitor assay	20	21-39	mg/dL
ACE	22	9-67	U/L
IgG4	37.7	4.0-86.0	mg/dL
Type 2 collagen Ab	17.2	<20	EU/mL

The rheumatology and hematology departments were consulted to help uncover the underlying etiology of his symptoms. Given the history of unknown fevers, orbital inflammatory syndrome (Figure 1), splenic infarcts, arthritis, chondritis, anemia with macrocytosis, bone marrow biopsy with vacuoles in myeloid and erythroid cells (Figure 3) and gene testing for VEXAS syndrome were recommended. His DNA analysis came positive for the *UBA1* p.Met41 mosaic mutation (exon 3, c.121A>G, pMet41Val). Moreover, given the constant pancytopenia, bone marrow gene testing was advised to check for *DNMT3A* and *TET2* mutations since he may be developing an MDS that could be responsive to current treatment options available on the market [8-9]. At this time, results are not yet available for these two mutations. The hematology department recommended continuing Eliquis after discharge for single-positive APLS studies and splenic infarcts [10-12]. He was also prescribed Bactrim® given the high-dose steroid taper received at discharge and a proton pump inhibitor.

## Discussion

The discovery of a new syndrome that does not meet diagnostic or classification criteria for other clinical conditions is challenging in many aspects. Based on recent studies, high-dose steroids were the only treatment that significantly ameliorated symptoms [1-2]. Our patient responded well to this management after failing other therapies, including disease-modifying antirheumatic drugs.

Additional experimental therapies have taken into account a combination of tocilizumab and methotrexate [13]. High serum levels of interleukin-6 (IL-6) were observed in patients with VEXAS syndrome. The effectiveness of tocilizumab (an IL-6 inhibitor) in other inflammatory conditions with high levels of IL-6, such as adult-onset Still's disease, may suggest a role in the treatment of VEXAS syndrome as well [14].

Other patients diagnosed with VEXAS syndrome have been shown to have characteristic somatic mutations in the genome that lead to loss of function (*TET2* and *DNMT3A*). These variations often result in the upregulation of pro-inflammatory cytokines such as IL-6 and IL-1, causing systemic inflammation [15]. Azacitidine was used with promising results in patients with suspected MDS who tested positive for the *DNMT3A* and *TET2* mutation, making hypomethylating agents efficacious in this subset of VEXAS patients [8-9].

Another study based out of France has shown how ruxolitinib (JAK2 inhibitor) and tofacitinib (JAK1/3 inhibitor) used for MDS are effective in patients with treatment-refractory VEXAS, even though this could be counterintuitive given that cytotoxic immunosuppressive agents can worsen cytopenias [16-17].

Allogenic hematopoietic stem cell transplant has been successful in a small subset of patients [18]; however, it is unclear how long patients can be on remission and their overall prognosis after transplant.

Our patient's management was complicated by single-positive APLS and splenic infarcts, requiring long-term anticoagulation. The team opted for direct oral anticoagulants (DOACs) instead of warfarin because the lab tests were significant only for B2GP1 IgM (Table 2). In these cases, it is acceptable to continue with DOAC alone unless complications arise, such as failure to respond to treatment [10-12].

It is also essential to consider that these patients are essentially immunocompromised and require vaccination such as Shingles, pneumococcal, and TDAP. However, the safety of new mRNA vaccines that have been used to control the surge of COVID-19 is unclear. In addition, our patient developed malaise and a cutaneous reaction soon after the injection due to unclear reasons.

## Conclusions

Our patient presented with typical features of VEXAS syndrome. Given the relatively new nature of this disease, diagnosis is still challenging for many health care providers. A few therapies are still undergoing investigation, but for now, a final cure is not yet available. Allogeneic hematopoietic stem cell transplantation is an option for patients refractory to current treatment and in a life-threatening clinical status. Complete remission was observed up to 67 months in one case, with one person dying from transplant complication; this approach could serve as a curative choice for the end-stage of this autoimmune condition. However, further studies are necessary to uncover other co-factors that affect the feasibility of this therapy, such as simultaneous gene mutations.

In regard to the use of new mRNA-based vaccines, it is wise to recommend that patients with VEXAS should abstain from receiving this new type of vaccination until more in-depth safety trials can be performed on their use for subjects with inflammatory conditions and autoimmune disorders such as VEXAS. Furthermore, it is paramount to avoid agents known to cause adverse reactions, unlike our patient who underwent full COVID-19 immunization, including the booster.

In conclusion, more studies are necessary to explore the curative options offered to this subgroup of patients. However, we are hopeful that with the advent of gene therapy and genetic analysis, those challenges can be overcome to provide a better life expectancy and quality of life to people affected by this rare condition.
